# Ferrous-Oxalate-Modified Aramid Nanofibers Heterogeneous Fenton Catalyst for Methylene Blue Degradation

**DOI:** 10.3390/polym14173491

**Published:** 2022-08-26

**Authors:** Lu Fu, Zhiyu Huang, Xiang Zhou, Liumi Deng, Meng Liao, Shiwen Yang, Shaohua Chen, Hua Wang, Luoxin Wang

**Affiliations:** Key Laboratory for New Textile Materials and Applications of Hubei Province, College of Materials Science and Engineering, Wuhan Textile University, Wuhan 430200, China

**Keywords:** ferrous oxalate, methylene blue, aramid nanofibers, heterogeneous Fenton catalyst, degradation

## Abstract

The heterogeneous Fenton system has drawn great attention in recent years due to its effective degradation of polluted water capability without limitation of the pH range and avoiding excess ferric hydroxide sludge. Therefore, simple chemical precipitation and vacuum filtration method for manufacturing the heterogeneous Fenton aramid nanofibers (ANFs)/ferrous oxalate (FeC_2_O_4_) composite membrane catalysts with excellent degradation of methylene blue (MB) is reported in the study. The morphology and structure of materials synthesized were characterized by scanning electron microscope (SEM), X-ray energy spectrum analysis (EDS), infrared spectrometer (FTIR), and X-ray diffraction (XRD) equipment. The 10 ppm MB degradation efficiency of composite catalyst and ferrous oxalate (FeC_2_O_4_) within 15 min were 94.5% and 91.6%, respectively. The content of methylene blue was measured by a UV-Vis spectrophotometer. Moreover, the dye degradation efficiency still could achieve 92% after five cycles, indicating the composite catalyst with excellent chemical stability and reusability. Simultaneously, the composite catalyst membrane can degrade not only MB but also rhodamine B (RB), orange II (O II), and methyl orange (MO). This study represents a new avenue for the fabrication of heterogeneous Fenton catalysts and will contribute to dye wastewater purification, especially in the degradation of methylene blue.

## 1. Introduction

Dye wastewater stemming from textile industries and dyestuff manufacturing is harmful to creatures on earth [1,2,3,4]. Many traditional methods have been used to remove the dye from the water, such as adsorption [5], chemical decolorization [6], and physical coagulation [7]. However, these dyes still exist in another state, without any degradation of the dye molecules. Thus, the chemical catalyst or photocatalyst as an efficient method for the degradation of dye has received more and more attention [8,9]. However, most catalysts have high costs due to the complex preparation process or equipment requirements. Simultaneously, the use of photocatalyst was limited by the catalytic conditions. Previous studies show that advanced oxidation processes (AOPs) can excite hydroxyl radicals to degrade organic pollutants into nontoxic small molecules in wastewater [10,11]. Compared with other oxidants, Fenton catalyst can degrade dye in the dark with a simple operation process, easy reaction, low operating cost, low equipment investment, and environmental friendliness. The Fenton method is a deep oxidation technology that utilizes the chain reaction between Fe^2+^ and H_2_O_2_ to catalyze the generation of hydroxyl radicals (•OH) [12,13,14]. In the reaction of the Fenton process, Fe^2+^ ions are oxidized by H_2_O_2_ to Fe^3+^ and one equivalent hydroxyl radical (•OH) is generated, as shown in Equation (1) [15,16,17]. In an acidic medium, Fe^3+^ can be reduced by H_2_O_2_ to establish a cycle of iron catalysts for subsequent activation. The mechanism is shown below in Equations (2) and (3) [18,19,20].

(1) $${\mathrm{Fe}}^{2+}+H_{2}O_{2}\to {\mathrm{Fe}}^{3+}+{\mathrm{OH}}^{-}+•\mathrm{OH}$$

(2) $${\mathrm{Fe}}^{3+}+H_{2}O_{2}\to {\mathrm{Fe}}^{2+}+{\mathrm{HO}}_{2}•+H^{+}$$

(3) $${\mathrm{Fe}}^{3+}+{\mathrm{HO}}_{2}•\to {\mathrm{Fe}}^{2+}+O_{2}+H^{+}$$

However, the homogeneous Fenton process is difficult to execute efficiently due to the limitation of the working pH range and the difficulty of separating the dissolved Fe^2+^ [21,22,23,24,25]. Thus, the heterogeneous Fenton process replaces the homogeneous Fenton system owing to its high activity, recyclability, and durability [26]. In recent years, catalytic fibers have been a very popular heterogeneous Fenton process for efficient decontamination of organic wastewater [27,28,29,30,31]. The combination of catalytic fibers and iron ion Fenton catalysts can solve the issue of poor recyclability of the powdered catalysts, resulting in a simplified process without precipitation and filtering [32]. Various fibers such as cellulose fiber [33], polyphenylene sulfide fiber [34], collagen fiber [35], and carbon fiber [36] have been applied as a carrier for immobilizing iron ion to prepare the Fenton catalyst.

The aramid fiber has received more and more attention due to its thermal stability, good chemical resistance, and excellent mechanical properties [37,38]. Thus, aramid nanofibers (ANFs) can keep their properties under •OH-containing conditions due to their excellent chemical stability. Simultaneously, the ANFs with a porous nanofiber network lamina structure could provide a specific surface area for catalyst decoration [39,40,41]. The ferrous oxalate (FeC_2_O_4_) can be synthesized via an inexpensively chemical precipitation method in mild conditions. Thus, ANFs/FeC_2_O_4_ can improve the chemical stability and degradation efficiency, and be easily separated from the solution without precipitation and filtration, compared with the powdered ferrous oxalate. However, little research has been reported on the synthesis and methylene blue degradation of the heterogeneous Fenton catalyst with FeC_2_O_4_ decorated ANFs.

In this study, the composite fiber membrane catalyst aramid nanofibers/ferrous oxalate (ANFs/FeC_2_O_4_) was synthesized as a heterogeneous Fenton catalyst via simple chemical precipitation and vacuum filtration method. To determine the optimal values of experimental variables for the degradation rate of MB solutions, a multivariate experimental design was performed, such as aramid nanofiber content, H_2_O_2_ concentration, initial pH, initial methylene blue (MB) concentration, and various structural dyes. In addition, the degradation rate maintains about an average of 92% after the ANFs/FeC_2_O_4_ was measured for five cycles, indicating excellent chemical stability. For the first time, the ANFs/FeC_2_O_4_ had been used in heterogeneous Fenton catalysis and degraded MB in dye wastewater.

## 2. Materials and Methods

### 2.1. Preparation of Aramid Nanofibers (ANFs) Suspension

Aramid nanofibers (ANFs) were obtained by Xinlin Tuo et al.’s method. Methoxypolyethylene glycols (mPEG Mw = 2000, 2.5 g) and calcium chloride (CaCl_2_, 9 g) were added to 100 mL n-methyl-2-pyrrolidone (NMP) and magnetically stirred at 100 °C. The reactor was reduced to 0 °C in an ice bath after the mixed solution dissolve. Then, 4.32 g p-phenylenediamine (PPD, purity 99%) was added to the above solution. Subsequently, terephthaloyl chloride (TPC, purity 99%) with molar ration at 1:1.007 (PPD:TPC) was added to the above solution and stirred at 70 °C. The mixture solution was shifted to deionized water under stronger shear. Finally, the ANFs suspension formed.

### 2.2. Preparation of Aramid Nanofibers (ANFs)/Ferrous Oxalate (FeC_2_O_4_) Composite Fiber Membrane

**Figure 1:**
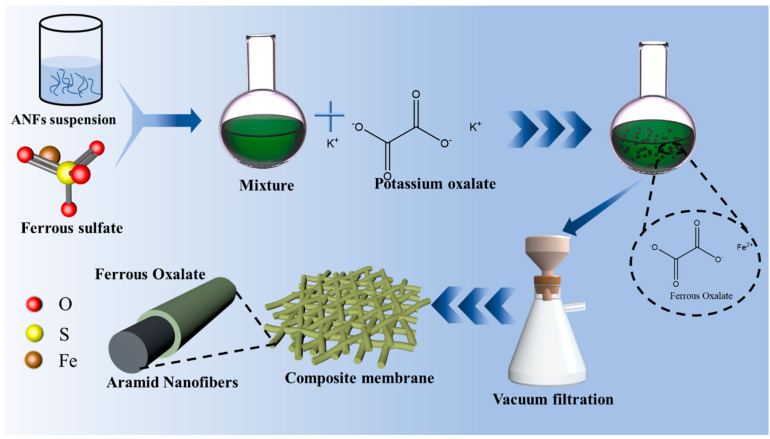
A schematic illustration of the preparation of ANFs/FeC_2_O_4_ composite fiber catalyst membrane.

Firstly, 0 g, 0.586 g, 1.005 g, 1.424 g, 2.261 g, and 4.774 g ferrous sulfate heptahydrate (FeSO_4_·7H_2_O) were dissolved in 100 mL ANFs suspension (0.13 g ANFs, 100 mL deionized water), respectively. The above mixture was stirred for 1 h at room temperature, respectively. Then, 0 g, 0.388 g, 0.666 g, 0.943 g, 1.498 g, and 3.163 g potassium oxalate (K_2_C_2_O_4_) were added to the above solution with stirring for 2 h, respectively. The composite catalyst membranes of ANFs/FeC_2_O_4_(0/100), ANFs/FeC_2_O_4_(30/70), ANFs/FeC_2_O_4_(20/80), ANFs/FeC_2_O_4_(15/85), ANFs/FeC_2_O_4_(10/90), and ANFs/FeC_2_O_4_(5/95) were obtained with 20 mL different proportions of aramid nanofibers and ferrous oxalate mixed solution via vacuum filtration method, respectively. Simultaneously, the FeC_2_O_4_ catalyst was obtained using 2.7801 g FeSO_4_·7H_2_O and 1.66 g K_2_C_2_O_4_ without ANFs by the same method as above. The schematic illustration is shown in Figure 1.

### 2.3. Experiment Procedure of Degradation of Methylene Blue

The methylene blue degradation process was simulated with 200 mL 10 ppm methylene blue solution with 150 ppm hydrogen peroxide and composite catalyst membrane in a Fenton reaction system at 20 °C. The pH was adjusted using 0.1 M H_2_SO_4_ and 0.1 M NaOH. A certain amount of sample solution was taken out of the reaction solution at specific time intervals. The absorbance of the sample solution was measured by UV-visible spectrophotometry (Shanghai Prism Technology Co., Ltd., Shanghai, China) at 664 nm wavelength. The methylene blue (MB) degradation efficiency can be calculated according to the following equation [42]:

Degradation efficiency (%) = 100% × (C_0_ − C_t_)/C_0_ (4)

C_0_ and C_t_ denote the concentration of the MB solution at 0 and t min, respectively.

### 2.4. Characterization

#### 2.4.1. Scanning Electron Microscope (SEM)

The surface morphologies of pure ANFs of ANFs/FeC_2_O_4_(20/80) catalysts were recorded on a field emission scanning electron microscope (SEM JEOL JSM-IT300A) at 15 kV voltage. A conductive layer was coated on the samples which were tested.

#### 2.4.2. BET Pore Size Analysis

The pore size distribution of pure ANFs and ANFs/FeC_2_O_4_(20/80) was analyzed at T = −195.8 °C by the BET (Micromeritics ASAP 2460), and the equilibration interval was 30 s.

#### 2.4.3. Fourier Transform Infrared Spectrometer

Infrared spectrogram of pure ANFs, pure FeC_2_O_4_, and ANFs/FeC_2_O_4_(20/80) was measured by FTIR (VERTEX70) attached ATR accessories to characterize a functional group of test samples with scanning varying from 500 cm^−1^ to 4000 cm^−1^. The six repeat scans were performed at 4.0 cm^−1^ intervals during the scanning range.

#### 2.4.4. X-ray Diffraction (XRD) Analysis

X-ray powder diffraction (XRD) patterns were recorded on an X-ray diffraction system (Empyrean, Holland) using Cu-Kα radiation (1.54 Å) in the scanning range from 10–60° (2θ) with steps of 0.02° (2θ).

#### 2.4.5. Measurement of Methylene Blue Concentration by UV-Vis Spectrophotometer

The methylene blue concentration was measured to characterize the degradation performance of the catalyst during the process of determining optimal degradation conditions. The concentration of methylene blue was measured using an ultraviolet spectrophotometer (A590 spectrophotometer, Shanghai, China) at 664 nm, which is the maximum absorbance of MB.

#### 2.4.6. X-ray Photoelectron Spectroscopy (XPS) Analysis

The surface elemental content of the fresh and used-five-times composite catalyst was investigated by the X-ray photoelectron spectroscopy (XPS, Thermo Scientific K-Alpha, Waltham, MA, USA) using Al Kα radiation.

## 3. Results and Discussion

### 3.1. ANFs/FeC_2_O_4_ Membrane Characterization

**Figure 2:**
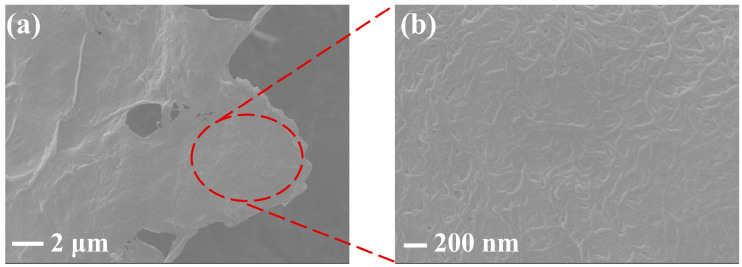
(**a**) SEM images of pure ANFs; (**b**) the magnified SEM image of pure ANFs.

**Figure 3:**
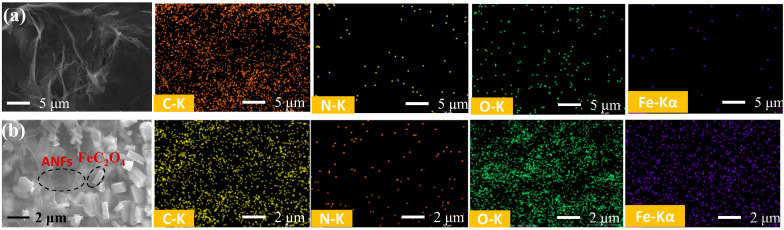
(**a**) SEM images of ANFs membrane, EDS element mapping images of ANFs; (**b**) SEM images of ANFs/FeC_2_O_4_ membrane, EDS element mapping images of ANFs/FeC_2_O_4_ membrane.

**Figure 4:**
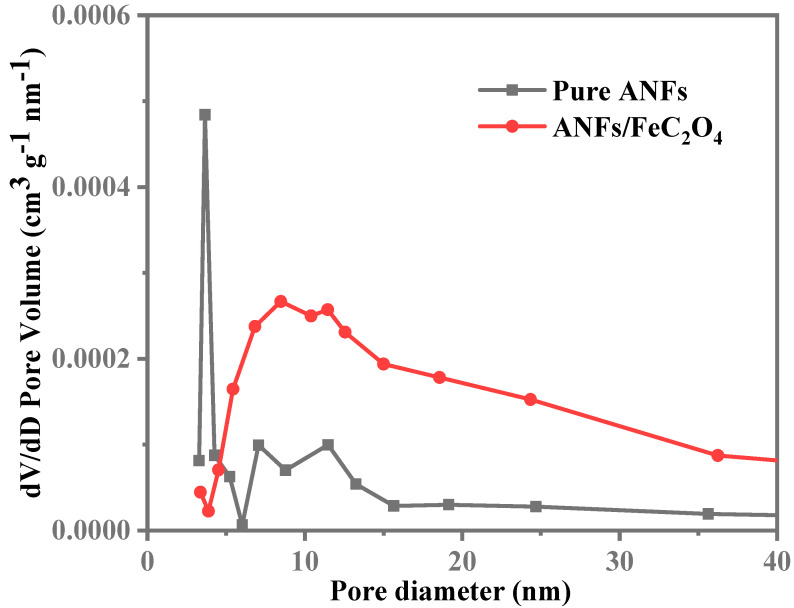
Pure ANFs and ANFs/FeC_2_O_4_ pore size distribution.

The fiber morphology of obtained ANFs was observed by scanning electron microscopy (SEM) as shown in Figure 2, and the diameter of the fiber is about 40 nm. The surface morphology and structure of the ANFs membrane and ANFs/FeC_2_O_4_(20/80) membrane were investigated by SEM. Shown in Figure 3a is the ANFs membrane with a nanofiber network lamina structure. The pore distribution can be seen clearly in Figure 4, the pore size of the composite FeC_2_O_4_ membrane was lager than that of the pure ANFs membrane. However, the content of pores of pure ANFs and ANFs/FeC_2_O_4_ membrane is very low, indicating it has a compact structure. The surface topography of ANFs membrane without obvious fiber morphology from the SEM images may be due to the strong intermolecular interactions leading to the fusion of ANFs and forming a compact structure during the formation of ANFs membrane via vacuum filtration [43,44]. The element distributions of the ANFs and ANFs/FeC_2_O_4_(20/80) membranes were observed using EDS element mapping, as shown in Figure 3. Compared with the ANFs membrane, obvious Fe element deposition could be observed in ANFs/FeC_2_O_4_(20/80) membrane. This phenomenon suggested that the FeC_2_O_4_ crystalline grains were formed on the fiber layer of the aramid nanofiber surface after the chemical deposition process, which meant that the ferrous oxalate was decorated on the ANFs successfully.

**Figure 5:**
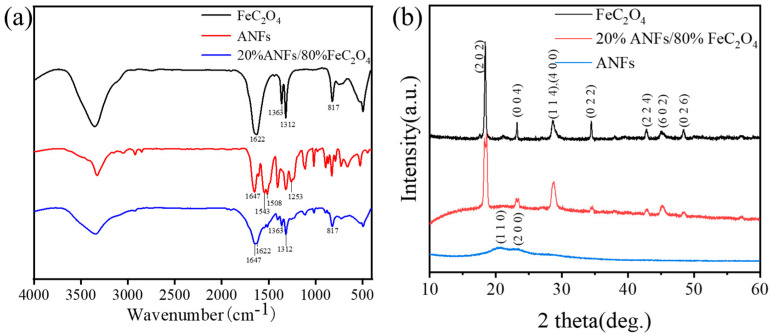
(**a**) FTIR spectra of FeC_2_O_4_, ANFs, and ANFs/FeC_2_O_4_. (**b**) XRD spectra of FeC_2_O_4_, ANFs, and ANFs-FeC_2_O_4_.

ATR FT-IR spectra were characterized to determine the chemical structure of the FeC_2_O_4_, ANFs, and ANFs/FeC_2_O_4_(20/80), as shown in Figure 5a. The peak at 1622 cm^−1^ shows the O-C-O asymmetric stretching vibration of C_2_O_4_^2−^, while the peak around 1363 cm^−1^ and 1312 cm^−1^ is the O-C-O symmetrical stretching vibration of C_2_O_4_^2−^ [34], and that at 817 cm^−1^ corresponds to the C_2_O_4_^2−^ O=C-O bending vibration [45]. The band at 1647 cm^−1^ can be attributed to the C=O stretching vibration [46,47]. The 1543 cm^−1^ and 1253 cm^−1^ can correspond to the N-H, C-N deformation coupling vibrations of ANFs. The band at 1509 cm^−1^ can be assigned to the C-C stretch of the aromatic nucleus [39,48]. These results show the FeC_2_O_4_ was decorated on the ANFs successfully.

XRD crystalline structure characterization of the ANFs, FeC_2_O_4_, and ANFs/FeC_2_O_4_(20/80) were shown in Figure 5b. The crystal structure of FeC_2_O_4_ is β-FeC_2_O_4_ (JCPDS No. 22-0635), and the peaks at 18.3°, 23.0°, 28.5°, 28.6°, 34.3°, 42.6°, 45.3°, and 48.2° are diffraction peaks of (2 0 2), (0 0 4), (1 1 4), (4 0 0), (0 2 2), (2 2 4), (6 0 2), and (0 2 6) lattice planes in β-FeC_2_O_4_. The peaks of ANFs at 20.5 and 23.3 are consistent with the (1 1 0) and (2 0 0) crystals in the poly-p-phenylene terephthamide crystal [49]. The diffraction pattern of the crystal anchored on the surface of the ANFs fiber is consistent with β-FeC_2_O_4_, and the peaks at 2θ values have no obvious deviation, indicating that the addition of ANFs would not change the structure of FeC_2_O_4_. Simultaneously, peaks of the ANFs/FeC_2_O_4_(20/80) catalyst were wider than that of FeC_2_O_4_, indicating that smaller FeC_2_O_4_ crystalline grains were decorated on the ANFs. The smaller size of FeC_2_O_4_ could improve the degradation performance of heterogeneous Fenton catalyst [50]. Thus, the ANFs/FeC_2_O_4_(20/80) catalyst was composed of ANFs and β-FeC_2_O_4_.

### 3.2. Influence of Experimental Conditions on Degradation Performance

#### 3.2.1. Influence of FeC_2_O_4_ Content and H_2_O_2_ Concentration in the Composite Film

**Figure 6:**
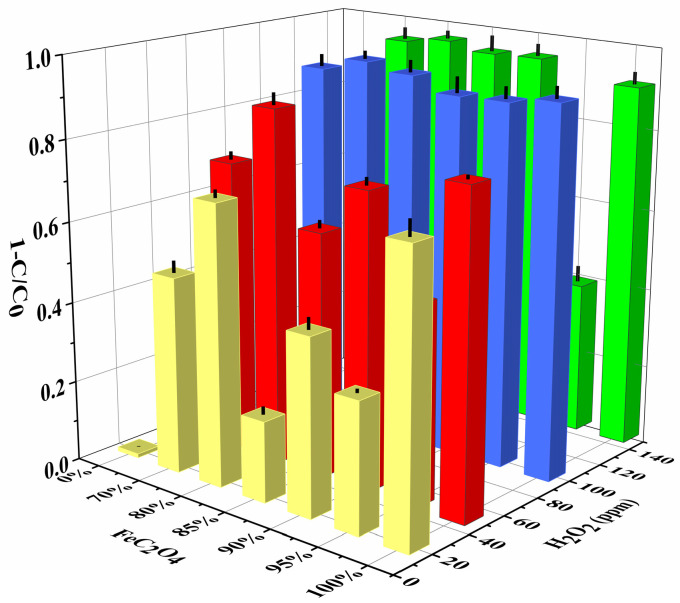
Effects of FeC_2_O_4_ content and H_2_O_2_ concentration on degradation performance.

The MB degradation performance of different ratio composite catalyst membranes was investigated, respectively. The pure ANFs had relatively poor degradation performance of MB, compared with the pure FeC_2_O_4_. However, when the FeC_2_O_4_ was decorated on the ANFs, they had better catalytic performance compared with the pure FeC_2_O_4_, as shown in Figure 6. The ANFs/FeC_2_O_4_(20/80) had the best degradation performance during ANFs/FeC_2_O_4_ composite catalyst with different proportions. The reason the degradation performance of MB decreased with the increase of ferrous oxalate may be that the ANFs did not have enough area to support the FeC_2_O_4_, resulting in the agglomeration of FeC_2_O_4_ affecting the degradation performance. Figure 5 shows the methylene blue degradation capacity improved with the accretion of H_2_O_2_ from 20 ppm to 100 ppm at an initial pH of 7. However, as the concentration of H_2_O_2_ increases, the degradation rate of MB is without significant change. The hydroxyl radicals (•OH) can be eliminated with the excessive addition of H_2_O_2_, resulting in the degradation rate without obvious increase, as shown in Equation (5) [51,52].

(5) $$H_{2}O_{2}+•\mathrm{OH}\to H_{2}O+{\mathrm{HO}}_{2}•$$

#### 3.2.2. Influence of Initial pH (pH_0_)

**Figure 7:**
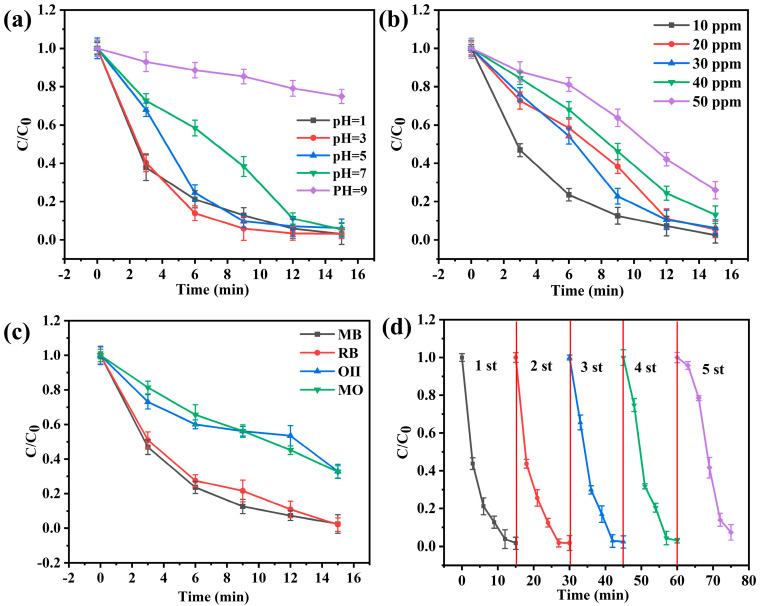
Influence of experimental conditions on degradation performance (FeC_2_O_4_ content = 80%, pH_0_ = 7, (MB) = 10 ppm, (H_2_O_2_) = 100 ppm, T = 20 °C): (**a**) initial pH; (**b**) MB concentration; (**c**) various dyes; (**d**) five periods of ANFs/FeC_2_O_4_ recycling.

The degradation efficiency of MB by heterogeneous Fenton’s process may be influenced by pH. In our study, the effect of pH from 1 to 9 on the degradation performance of MB was explored. The MB degradation efficiency did not show an obvious difference when pH = 1, 3, 5, and 7 within 15 min (Figure 7a), indicating the catalyst is not limited to acidic conditions. Compared with the homogeneous Fenton catalyst (pH range = 3–4), the heterogeneous Fenton catalyst improves pH working range [53]. The final MB degradation efficiency did not show reduction at pH 1. However, the MB degradation velocity decreased slightly at pH 1. The hydrogen peroxide solvates a proton to bring into being an oxonium ion (H_3_O^2+^), which may be the reason for the decrease of degradation performance at pH 1. The hydrogen peroxide with electrophilicity due to the presence of oxygen ions results in the stability improvement of hydrogen peroxide and reduced reactivity with ferrous ions [54]. As reported by other studies [34,55], Fenton’s catalyst loses its degradation performance at low pH due to the Fe^3+^ being attacked by the H_2_O_2_, and inactivated into Fe^4+^, according to Equation (6). However, the C_2_O_4_^2−^ of ANFs/FeC_2_O_4_ can chelate Fe^3+^ to a certain extent to prevent Fe^3+^ from peroxidation. Thus, the ANFs/FeC_2_O_4_ can degrade dyes at low pH and expand the pH working range. At pH = 9, the MB degradation is drastically reduced, which may be due to the low dissolution capability of iron species [56,57]. The pH_0_ of the heterogeneous degradation process with the ANFs/FeC_2_O_4_(20/80) catalyst was shown to have an optimum value of 7 for the degradation of MB.

(6) $${\mathrm{Fe}}^{3+}+H_{2}O_{2}+H^{+}\to {\mathrm{Fe}}^{4+}+•\mathrm{OH}+H_{2}O$$

#### 3.2.3. Influence of Initial MB Concentration

The effect of initial methylene blue concentration on degradation performance was investigated by changing the concentration from 10 to 50 ppm, as shown in Figure 7b. The degradation velocity decreases with increasing initial methylene blue concentration. However, the ultimate degradation amount of MB improved as the initial MB concentration increased. As the MB concentration increases, the active site is surrounded by enough MB molecules, and the activated hydroxyl groups have more opportunities to attack the MB for degradation, whereas the increase in the degradation amount of MB lags the increase in the initial MB concentration, resulting in the decrease of the degradation efficiency. It can be seen that the degradation efficiency of MB gradually decreased with the increase of the initial concentration of MB. When the initial concentration is 10 ppm, the composite catalyst has higher degradation efficiency. Based on the maintenance of other conditions in the solution, the concentration of 10 ppm methylene blue was used as a fixed value in subsequent experiments.

#### 3.2.4. Influence of Different Dyes

As shown in Figure 7c, the degradation rate of various dyes was assessed by using different models of dyes. According to the different types of charges carried on the surface of the dye’s particles, organic dyes can be mainly classified into two categories: cationic dyes (methylene blue, MB; rhodamine B, RB; methyl orange, MO; anionic dyes (orange II, OII). Both methyl orange and orange II are azo dyes, and the relatively low degradation rate may be due to the presence of azo and aryl groups. It can be seen that the cationic and non-azo dyes have a higher degradation rate than the anions and azo dyes. The reason for the ANFs/FeC_2_O_4_ composite catalyst membrane providing better degradation performance of cationic dyes may be due to the ANFs having negativity [58,59,60] which can faster adsorption of cationic dye molecules, corresponding to Mohammed’s previous report [61]. However, various structures and types of dyes can be efficiently degraded by the ANFs/FeC_2_O_4_ composite membrane catalyst. In this experiment, methylene blue is a typical cationic non-azo dye, it is selected as a representative degradable dye.

#### 3.2.5. Influence of Cycle Performance

**Figure 8:**
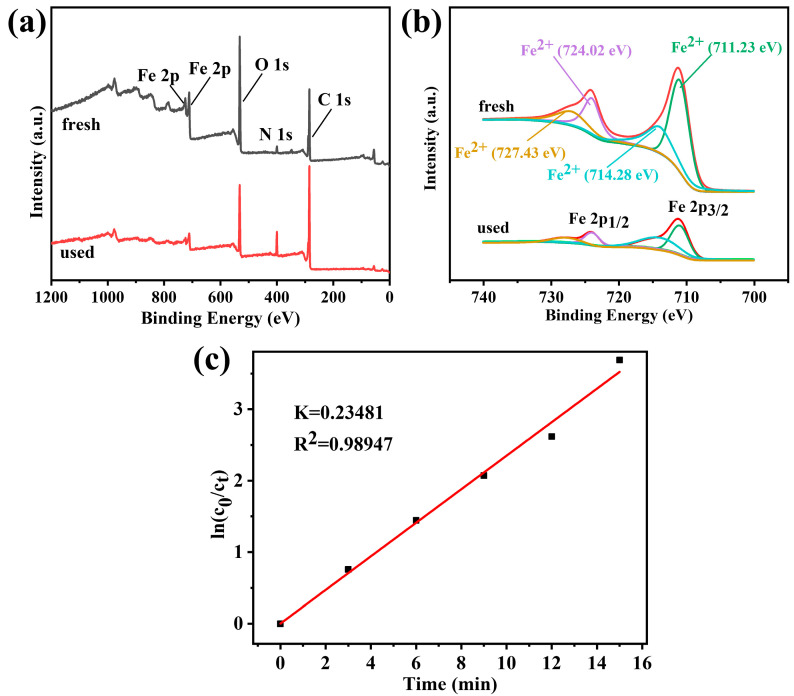
(**a**) The XPS survey spectral of the fresh and used-five-times composite catalyst; (**b**) spectra of Fe 2p; (**c**) pseudo-first-order kinetics of degradation of MB (FeC_2_O_4_ content was 80%, pH_0_ = 7, MB content was 10 ppm, H_2_O_2_ content was 100 ppm, T = 2 °C).

As shown in Figure 7d, the cycle stability of ANFs/FeC_2_O_4_ survived five periods of the cyclic degradation experiment. During the cycling experiment, the used catalyst was thoroughly rinsed with deionized water, and vacuum dried as the initial preparation process. The degradation performance is not significantly different from the first in the second cycle process. However, starting from the third cycle of the process, the degradation performance of the process was reduced slightly. As illustrated in Figure 8, compared with the fresh catalyst, the content of iron in the catalyst after being reused five times was decreased. The reason for the decrease in degradation rate may be due to the trace abscission of active sites in the ANFs/FeC_2_O_4_ during flushing. The Fe 2p narrowband XPS spectrum (Figure 8b) of the catalyst was scanned over the binding energy range of 740 eV to 700 eV. The simulated peaks at 727.43 eV and 724.02 eV can be confirmed as Fe^2+^ characteristic peaks in Fe 2p1/2, and the simulated partial peaks at 711.23 eV and 714.28 eV can be confirmed as Fe^2+^ characteristic peaks in Fe 2p3/2, respectively. The curve of the used catalyst is similar to a fresh catalyst, and there is no obvious Fe^3+^ characteristic peak. The decrease in peak intensity is due to the decrease in relative element content. So, the iron elements on the surface of the catalyst before and after the reaction are mainly Fe^2+^ particles. During the degradation process, Fe^2+^ on the catalyst surface is oxidized in the hydroxyl radical formation reaction. However, Fe^3+^ characteristic peaks were not observed in the Fe 2p narrow-band XPS spectrum. The formation of Fe^3+^ can be reduced to Fe^2+^ and reactivated as an active site. Eventually, the catalytic performance of the ANFs/FeC_2_O_4_ decreased slightly. In a word, the ANFs/FeC_2_O_4_ has high catalytic stability and good recycling performance in the cyclic degradation process.

#### 3.2.6. The Kinetics of Degradation of MB

According to Section 3.2.1–Section 3.2.4, Fenton catalyzed degradation results, based on the Langmuir–Hinshelwood model [62,63] of the photocatalytic kinetics of Fenton catalyst for MB degradation, were investigated. During photocatalytic degradation, when the molecular adsorption reaches equilibrium, the pseudo-first-order kinetic equation can be expressed as Equation (7). If the degradation of methylene blue by Fenton catalyst is a pseudo-first-order reaction, the above formula can be simplified to Equation (8). After integration, Equation (9) is obtained.

(7) $$v=-\frac{dC_{t}}{dt}=k_{r}K_{\mathrm{ad}}C_{t}/\left(1+K_{\mathrm{ad}}C_{t}\right)$$

(8) $$v=-\frac{dC_{t}}{dt}=k_{r}K_{ad}C_{t}=KC_{t}$$

(9) $$\ln\left(\frac{C_{0}}{C_{t}}\right)=Kt$$

$k_{r}$ is the reaction rate constant, $K_{\mathrm{ad}}$ is the adsorption equilibrium constant, $C_{t}$ represents the concentration of MB at *t*, and $C_{0}$ represents the MB concentration after adsorption and desorption at equilibrium, *K* represents the pseudo-first-order rate equation constant.

The fitting curve of the pseudo-first-order kinetics of MB catalyzed by the composite catalyst is shown in Figure 8c. The fitted linear correlation coefficient of the composite Fenton catalyst was 0.98947, indicating that the catalytic degradation of MB by the catalyst conformed to a pseudo-first-order kinetic model.

### 3.3. Degradation Mechanism

**Figure 9:**
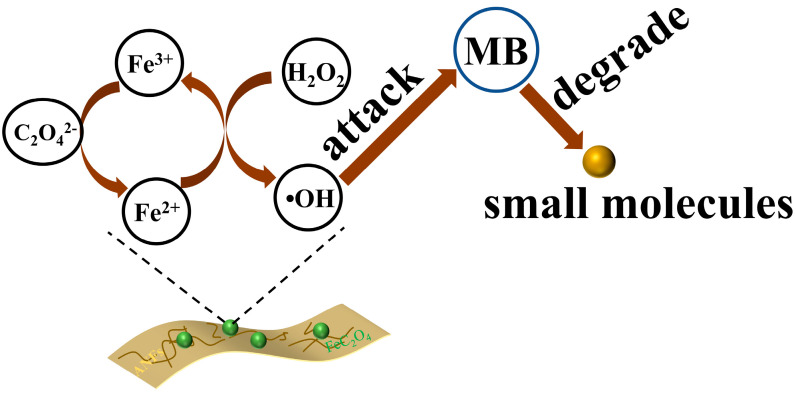
The MB degradation process of composite Fenton catalyst film.

**Figure 10:**
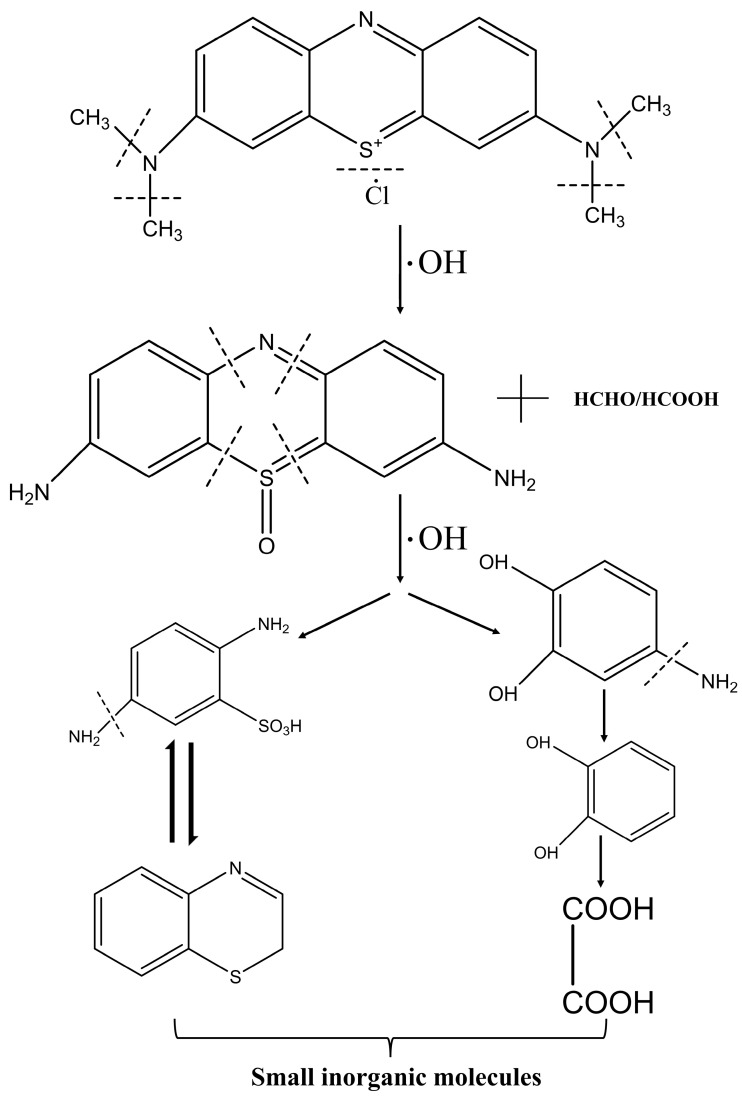
Schematic diagram of the degradation mechanism of methylene blue.

The ANFs/FeC_2_O_4_ and H_2_O_2_ is a heterogeneous Fenton system. During the degradation process, the active sites on the surface of ANFs/FeC_2_O_4_ can effectively excite hydroxyl radicals, as shown in Equation (1), and the hydroxyl radicals can effectively degrade methylene blue molecules. During the excitation of hydroxyl radicals by ferrous oxalate, Fe^2+^ is oxidized to form Fe^3+^, and, at the same time, C_2_O_4_^2−^ can reduce the formed Fe^3+^ again and reactivate the active sites on the catalyst surface that were deactivated due to the excitation of hydroxyl radicals. Thus, methylene blue molecules are degraded into small molecules by the attack of hydroxyl radicals (Figure 9). During the dissolution of methylene blue molecules, Cl^−^ is ionized and exists in a dissociated state. As shown in Figure 10, the N-CH_3_ bond in the methylene blue molecule is preferentially broken by the attack of •OH, then the methyl groups are oxidized to formaldehyde and formic acid. The hydroxyl groups continue to attack the C-S and C-N bonds to produce unstable organic matter, and the MB is eventually degraded into small molecules [64,65,66]. The above heterogeneous Fenton degradation process shows the ANFs/FeC_2_O_4_ composite membrane catalyst with excellent MB degradation performance, reusability, and catalytic stability. The above results (Figure 6) show that the ANFs/FeC_2_O_4_ have an excellent ability to degrade methylene blue compared with the pure ANFs and FeC_2_O_4_. The ANFs can effectively provide a loading area for ferrous oxalate to prevent the agglomeration of ferrous oxalate, resulting in more active sites being exposed. Thus, the MB degradation performance of heterogeneous Fenton ANFs/FeC_2_O_4_ was improved.

## 4. Conclusions

In summary, we demonstrated that the FeC_2_O_4_ could be decorated on the ANFs successfully due to the microfibers of ANFs, with excellent methylene blue degradation performance. The •OH can be effectively excited from H_2_O_2_ by the active site of ANFs/FeC_2_O_4_ composite membrane catalyst to degrade MB under suitable conditions. The ANFs/FeC_2_O_4_ composite membrane catalyst provides better MB degradation performance compared with the pure FeC_2_O_4_. More importantly, the ANFs/FeC_2_O_4_ composite membrane catalyst could be simply separated from methylene blue solution without precipitation and filtration, compared with the powder ferrous oxalate, reducing tedious and time-consuming separation processes. The ANFs/FeC_2_O_4_(20/80) composite catalyst membrane with 100 ppm H_2_O_2_ at pH = 3 degrade 10 ppm MB was the optimal degradation condition which was explored through a series of degradation experiments. The composite catalysts with excellent degradation methylene blue performance were proven, indicating the composite catalysts can be used in methylene blue wastewater treatment. The preparation of ANFs has achieved industrial production and the composite membrane was obtained by simple chemical precipitation and vacuum filtration method. Simultaneously, the main component of the catalyst is ferrous oxalate, which is synthesized from low-valent ferrous sulfate and potassium oxalate at room temperature without any special experimental equipment at a low cost. Thus, these results show the composite catalyst has significant potential in practical methylene blue wastewater treatment and industrial production is expected.

## Data Availability

The data presented in this study are available in this study.
